# Estimating Malaria Incidence through Modeling Is a Good Academic Exercise, but How Practical Is It in High-Burden Settings?

**DOI:** 10.4269/ajtmh.20-0120

**Published:** 2020-03-02

**Authors:** Yazoume Ye, Andrew Andrada

**Affiliations:** ICF, Rockville, Maryland

There is global interest in frequent reliable estimates of the malaria burden, especially at the country and subnational levels, to determine progress in malaria control efforts. Traditionally, these estimates were obtained mostly from national household surveys such as demographic and health surveys and malaria indicator surveys. However, routine health information systems (RHIS) are increasingly being used to refine these estimates, especially at the subnational levels and during periods between surveys. Currently, the WHO provides malaria burden estimates annually through the *World Malaria Report*, an important information source for the global malaria community to track efforts and progress. The 2018 estimates were generated through a model-based geostatistical framework, which converts *Plasmodium falciparum* parasite rate (PfPR) data to malaria incidence in high-burden countries and converts case data captured through surveillance to national incidence and PfPR estimates in lower burden countries.^1,2^ Because these estimates rely on country RHIS data and modeling, there is an inherent degree of uncertainty, and the results, in some cases, are challenged by countries.

In addition to these methods, other approaches including time-series analysis, such as auto-regressive integrated moving averages,^3,4^ have been used on RHIS data to estimate the malaria burden and understand the effects of malaria interventions. Programs are increasingly focusing efforts on surveillance as a key data source to routinely monitor progress and inform program implementation. As RHIS improve in malaria-endemic countries, routine data are becoming more available and accessible to generate estimates with real-time data. However, these data are sensitive to several biases related to population access to health care, care-seeking behavior, malaria testing rates, and reporting completeness. The article, “A robust estimator of malaria incidence from routine health facility data” by Thwing et al.^5^ in this issue, aimed to address these issues using an algebraic method that relies on a number of assumptions to improve malaria incidence estimates and quantify commodity needs. The authors applied their method on RHIS data to generate incidence estimates and estimate gaps in malaria testing and treatment needs from *World Malaria Report*–reported incidence rates for Guinea, Mozambique, and all of sub-Saharan Africa.

The goal for developing this method was to provide national malaria control program (NMCP) staff and their partners a tool to interpret routinely reported surveillance data without needing a background in statistics to understand the results, and to provide reliable burden estimates to allow for comparison between districts or regions, and to evaluate trends over time. The authors presented their approach as a more robust method to adjust crude incidence rates for potential biases. However, to better interpret these results, the underlying assumptions need to be understood. Furthermore, although their approach is intended to help inform the NMCP strategy, it will require time and financial resources to introduce this model and for NMCP staff to understand it and develop actionable strategies. In addition, as the authors noted, their method has several limitations, which requires unbiased data and correct classification of key data elements.

There is a need to accurately determine the burden of malaria to measure progress. Although the method developed by Thwing et al.^5^ is designed to help NMCPs, it generates estimates with a level of uncertainty that requires some level of statistical skills to interpret, and these skills are not always present in NMCPs. These models and methods are a good academic exercise to improve estimates and streamline NMCP actions; however, in the context of high transmission, the precision of malaria incidence estimates is unlikely to affect NMCP strategies in the short term. Rather, in settings with high malaria incidence and universal coverage targets, the use of crude incidence estimates from malaria surveillance systems may be sufficient for guiding the NMCP strategies. The situation might be different in low-transmission settings, where precise estimates of malaria incidence will be required to monitor progress toward elimination.

Estimates from models are deterministic and rely heavily on a country’s surveillance system coverage and quality of data. Instead of relying on models, countries should be supported in strengthening their routine systems. According to a recent malaria surveillance system landscaping across sub-Saharan Africa, common gaps included the lack of surveillance coverage in remote areas and across the private healthcare sector, inadequate health information infrastructure to capture and manage high-quality case-based data, poor integration of data within the NMCPs, limited capacity for data visualization, and lack of quality and timely data for decision-making.^6^ Focusing efforts on addressing these gaps to strengthen surveillance systems and RHIS will lead to improved data quality, expanded coverage, and increased precision of malaria incidence estimates. Similarly, as countries update their case management practices, expand access, and standardize reporting, more suspected cases will be tested, and positive cases treated, providing improved and more current data. As malaria transmission declines, a country’s surveillance system becomes critical, and a strong reactive and active case detection system is needed to ensure progress toward malaria elimination. Malaria elimination classification is based on these systems, which are more sensitive to stochastic incidence than modeling.

Models provide insights into the effectiveness of interventions and spatiotemporal trends of the malaria burden. However, because of the underlying assumptions these models are built on, their use may be less practical from the program perspective than use of surveillance data. Devoting resources to strengthen routine surveillance systems that generate these data will have greater utility to NMCPs, improve reliability of the data, and encourage data use. Developing these systems in collaboration with countries can garner country ownership of the data, simplify data analysis, inform NMCP strategies (without need for mathematical and statistical modeling), build capacity within the country, and promote self-reliance.
